# Mucocele of the appendix: case report of a rare disease with changing diagnostic-therapeutic behavior

**DOI:** 10.1093/jscr/rjae397

**Published:** 2024-06-04

**Authors:** Mauro Giambusso, Giovanni Salvatore Urrico, Giovanni Ciaccio, Francesco Lauria, Sara D’Errico

**Affiliations:** Department of General Surgery, Vittorio Emanuele Hospital, 93012, Gela, Italy; Department of Pathological Anatomy, Sant’Elia Hospital, 93100, Caltanissetta, Italy; Department of General Surgery, Sant’Elia Hospital, 93100, Caltanissetta, Italy; Department of General Surgery, Vittorio Emanuele Hospital, 93012, Gela, Italy; Department of General Surgery, Vittorio Emanuele Hospital, 93012, Gela, Italy

**Keywords:** appendix, mucocele, LAMN

## Abstract

Mucinous appendicular neoplasms are a rare and heterogeneous group of tumors, whose treatment may vary based on histologic features and extent. We present a case of low-grade appendiceal mucinous neoplasm mimicking an acute appendicitis scenario. The patient underwent appendectomy along with resection of the caecal fundus. Choosing the correct treatment according to the case by following current guidelines is crucial to avoid under- or overtreatment.

## Introduction

Appendiceal mucocele (AM) is defined as a progressive distension of the appendix, of obstructive origin, due to intraluminal accumulation of mucinous material.

It has been reported to have an incidence of 0.2–0.3% in all appendix specimens [1], with a higher frequency as age increases [2]. In case of spontaneous or iatrogenic perforation, AM can lead to pseudomyxoma peritonei (PMP), a rare tumorous disease with various grades of malignancy characterized by the spreading of mucinous implants in the peritoneal cavity [3].

The term mucocele (or mucinous cystadenoma) is an umbrella term and is recently discouraged by some authors, though it is still in use by radiologists and surgeons [4].

We present a case of low-grade appendiceal mucinous neoplasm (LAMN) diagnosed on histopathological examination after surgery.

The aim of this manuscript is to underline the correct preoperative diagnostic workflow and the surgical management.

Informed consent was received from the patient. The work has been reported in line with the SCARE 2020 criteria [5].

## Case report

**Figure 1:**
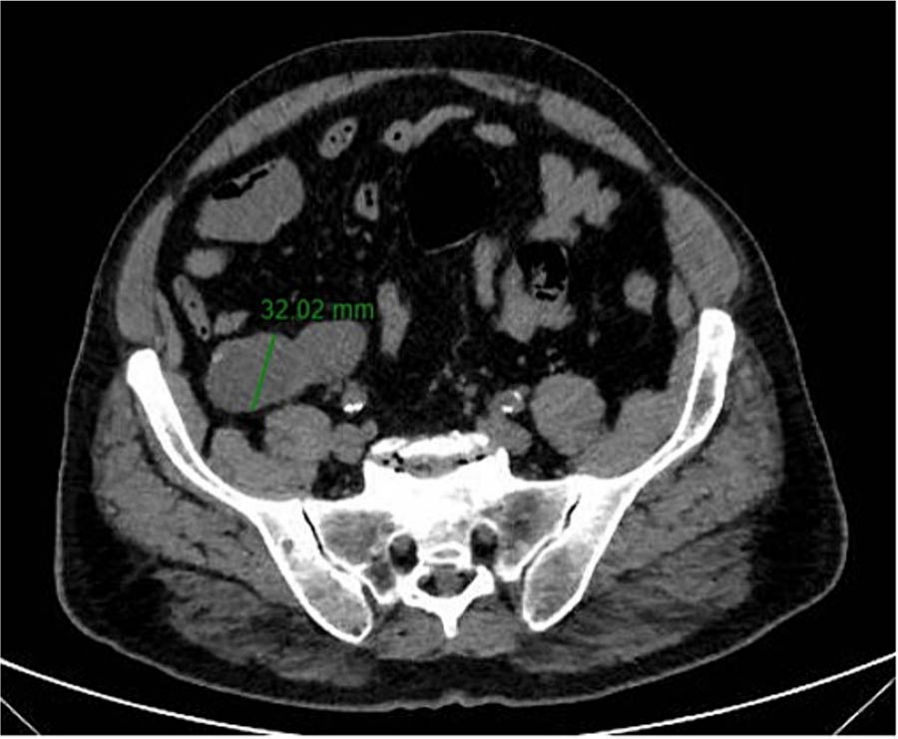
Axial CT-scan highlighting the AM.

**Figure 2:**
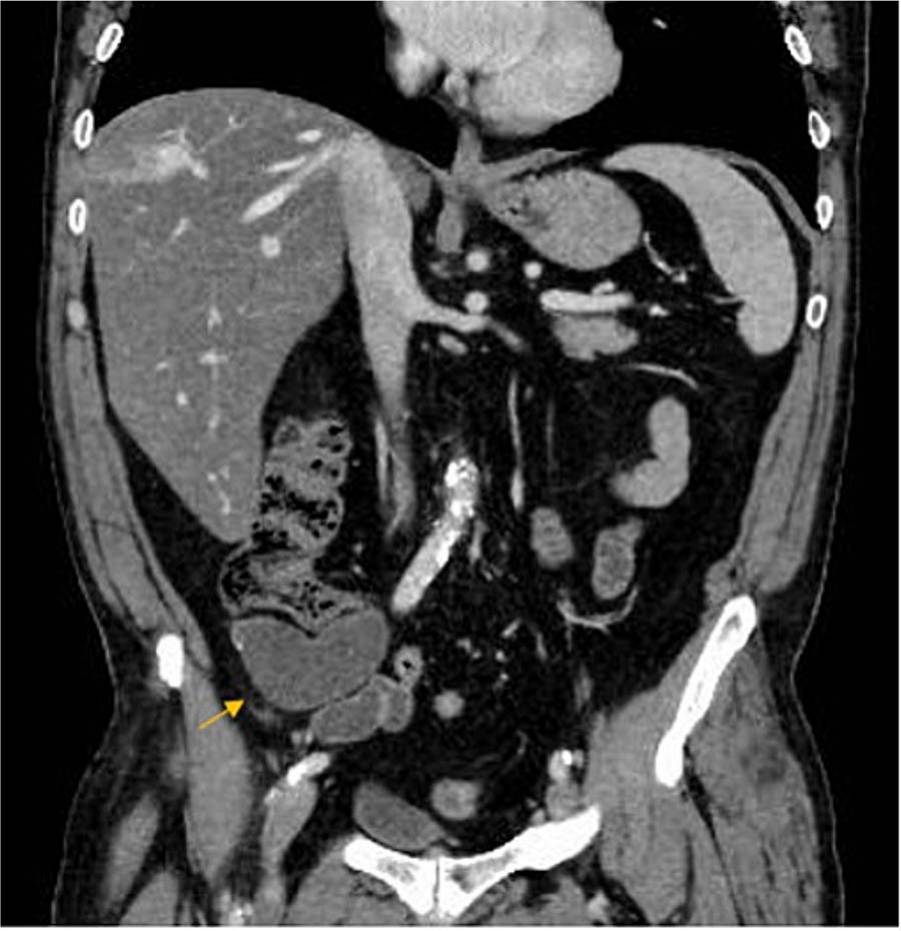
AM on coronal CT-scan.

A 67-year-old man presented to our emergency department for abdominal pain occurred about 3 days ago. Past medical history included ischemic heart disease, type II diabetes mellitus, chronic obstructive pulmonary disease, obstructive sleep apnea syndrome, smoking. No history of previous surgery. Laboratory markers showed hemoglobin 13.3 g/dL, leukocytes 8.9 × 10^9^/L, C-reactive protein 11 mg/L. CT scan detected a 7.5 × 3.2 cm diameter suprafluid tubular neoformation in continuity with the caecal fundus, compatible with AM (Figs 1 and 2). A contextual focal wall thickening of 1.1 × 0.5 cm in diameter with slender calcifications was also appreciated. No repetitive parenchymal or peritoneal nodularity had been documented. Colonoscopy showed a rosy and regular mucosa in all colic segments explored up to the cecum, with no detectable pathological lesions.

**Figure 3:**
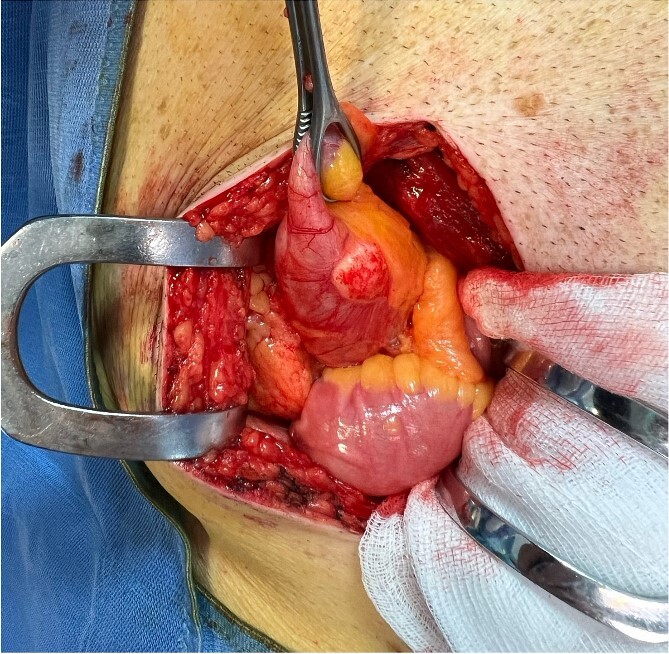
Intraoperative findings showing pathologic appendix and whitish neoformation in site compatible with mucocele.

**Figure 4:**
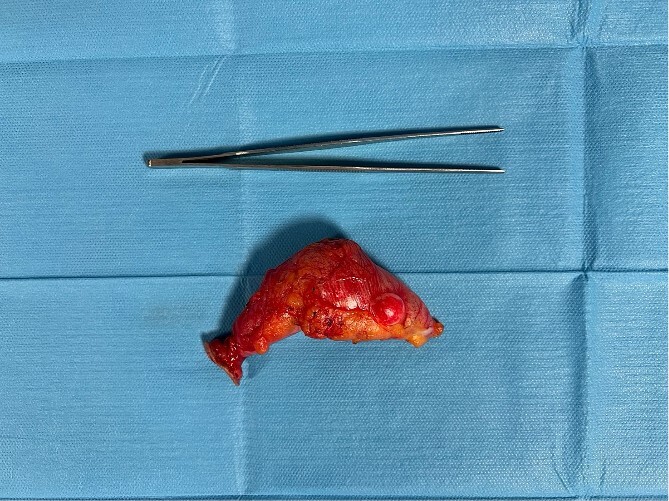
Surgical piece illustrating the appendicular mucocele after stapled appendectomy and tangential resection of the cecum base.

The patient underwent explorative laparotomy after right pararectal incision. An increased size and consistency appendix was found, with concomitant single whitish neoformation of about 1 cm maximum diameter, as suspected on CT scan (Fig. 3). Neither mucin nor repetitive peritoneal lesions were objectifiable on either abdominal inspection or palpation (PCI score = 1) [6]. A stapled section of the appendix with partial inclusion of the wall of the caecum was performed (Fig. 4). The patient was discharged 4 days after the surgery, no postoperative complications occurred.

**Figure 5:**
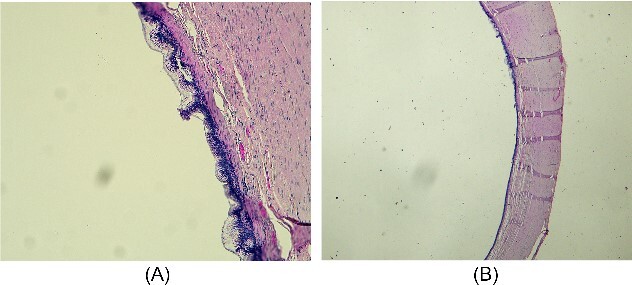
Microscopic appearance of mucinous cystadenoma of the appendix. (A) Proliferative and partially papillary configuration of the mucosa; haematoxylin and eosin, magnification ×20. (B) Evidence of flattened mucosa accompanied by atrophy, fibrosis and chronic inflammation of the underlying wall; haematoxylin and eosin, magnification ×10.

The resected specimen consisted of 8-cm-long appendix with enlarged lumen, comprising a cystic neoformation with a maximum diameter of 1.8 cm and mucinous content, partially disepithelized and sometimes flattened mucinous and cubic epithelial lining, in the absence of atypia. A chronic lymphocytic inflammatory infiltrate of the bowel wall was associated. The caecum resection margin was free of disease. Histopathological findings were compatible with the diagnosis of LAMN according to the International Modified Delphi Consensus Process classification [7] (Fig. 5A–B). At 6-month follow-up, the patient resulted in good condition.

## Discussion

AM was first described by Rokitansky in 1842. After more than 150 years, the term AM seems to be obsolete and a source of many misunderstandings in the scientific field. In this regard, to make it clear, the classification of the International Modified Delphi Consensus Process has been universally accepted.

Appendicular neoplasms are rare, accounting for only 0.5% of digestive tract tumors [8]. The most common are epithelial and neuroendocrine tumors. Epithelial tumors are divided into non-mucinous (adenoma, adenocarcinoma) and mucinous neoplasms (LAMN, HAMN, mucinous adenocarcinoma). Up to 1% of appendectomy specimens are diagnosed with LAMN, tumors of uncertain malignant potential mainly affecting women over 60 years of age [9].

Due to clinical heterogeneity, preoperative diagnosis of LAMN is still challenging. It can be asymptomatic, being diagnosed incidentally or may be presented by mimicking appendicitis symptoms. In advanced stages, symptoms may include chronic abdominal pain, urinary retention, constipation or new onset umbilical or inguinal hernias caused by increased abdominal pressure. Early and advanced symptoms were present in this patient, with evidence of appendicular inflammation but no accumulation of mucinous ascites in the peritoneum.

The radiological gold standard is a CT scan with or without intravenous and oral contrast agent. Preoperatively contrast-enhanced CT has a 95% sensitivity in detecting appendiceal tumor in patients with symptoms of appendicitis [10].

MRI is recommended for both staging and follow-up of appendiceal tumors due to its higher sensitivity and specificity than CT in detecting peritoneal spread of disease [11].

Laboratory findings are non-specific and include anemia or elevated tumor markers [12]. Tumor mass perforation causes an inflammatory response similar to perforated appendicitis due to the expulsion of mucus [13]. In the case we reported, the lesion was not perforated and the inflammation rates were within the normal range.

Sometimes, in the suspicion of a mucinous epithelial tumor of the appendix, it’s useful to perform a preoperative colonoscopy to prevent an unnecessary caecectomy. In our experience, no caecum lesions were found at preoperative colonoscopy.

Resection of the appendix and caecum fundus with relative mesentery is the preferred approach [14]. Right colon resection with ileocolic lymph nodes dissection is not associated with improved prognosis over appendectomy alone [15]. Laparotomy is the recommended surgical technique due to its lower risk of accidental opening of the specimen [16]. A LAMN removed intact is the surgical goal to consider the patient cured. A disrupted specimen may frequently result in pseudomyxoma peritonei [17].

Treatment of appendicular neoplasms can vary considerably depending on both histotype and extent of pathology [18]. Addressing the therapeutic strategy in the correct way is of paramount importance for the surgeon. In our case report, a patient suffering from LAMN in the absence of PMP signs underwent appendectomy with caecal fundus resection, following the current recommendations in the literature.
